# Causal inference shapes crossmodal postdiction in multisensory integration

**DOI:** 10.1038/s41598-026-36884-6

**Published:** 2026-02-21

**Authors:** G. Günaydın, J. K. Moran, T. Rohe, D. Senkowski

**Affiliations:** 1 Department of Psychiatry and Neurosciences, Charité Campus Mitte (CCM), Charité - Universitätsmedizin Berlin, Corporate Member of Freie Universität Berlin and Humboldt-Universität Zu Berlin, Charitéplatz 1, 10117 Berlin, Germany; 2 Bernstein Center for Computational Neuroscience Berlin, Humboldt-Universität Zu Berlin, Philippstr. 13, Haus 6, 10115 Berlin, Germany; 3 Department of Psychology, Humboldt-Universität Zu Berlin, 10099 Berlin, Germany; 4 Einstein Center for Neurosciences Berlin, Berlin Institute of Health, Charité – Universitätsmedizin Berlin, Corporate Member of Freie Universität Berlin, Humboldt-Universität Zu Berlin, Charitéplatz 1, 10117 Berlin, Germany; 5 Sensory Analytics and Technologies, Fraunhofer Institute for Process Engineering and Packaging (IVV), Giggenhauser Str. 35, 85354 Freising, Germany; 6 Institute of Psychology, Friedrich-Alexander-Universität, 91054 Erlangen-Nürnberg, Germany

**Keywords:** Neuroscience, Psychology, Psychology

## Abstract

In our environment, stimuli from different sensory modalities are initially processed within a temporal window of multisensory integration spanning several hundred milliseconds. During this window, stimulus processing is influenced not only by preceding and current information, but also by input that follows the stimulus. The computational mechanisms underlying crossmodal backward processing, which we refer to as crossmodal postdiction, are not well understood. We examined crossmodal postdiction in the Illusory Audiovisual (AV) Rabbit and Invisible AV Rabbit Illusions, in which postdiction occurs when flash-beep pairs are presented shortly before and shortly after a single flash or a single beep. We collected behavioral data from 32 participants and fitted four competing models: Bayesian Causal Inference (BCI), forced-fusion, forced-segregation, and non-postdictive BCI. The BCI model fit the data well and outperformed all other models. Building on previous findings that demonstrate causal inference during non-postdictive multisensory integration, our results show that the BCI framework can also explain crossmodal postdiction phenomena. Our findings suggest that the brain performs causal inference not only across concurrent sensory inputs but also across temporal windows, integrating information from past, present, and subsequent events across modalities to construct a unified percept.

**Supplementary Information:**

The online version contains supplementary material available at 10.1038/s41598-026-36884-6.

## Introduction

**Fig. 1:**
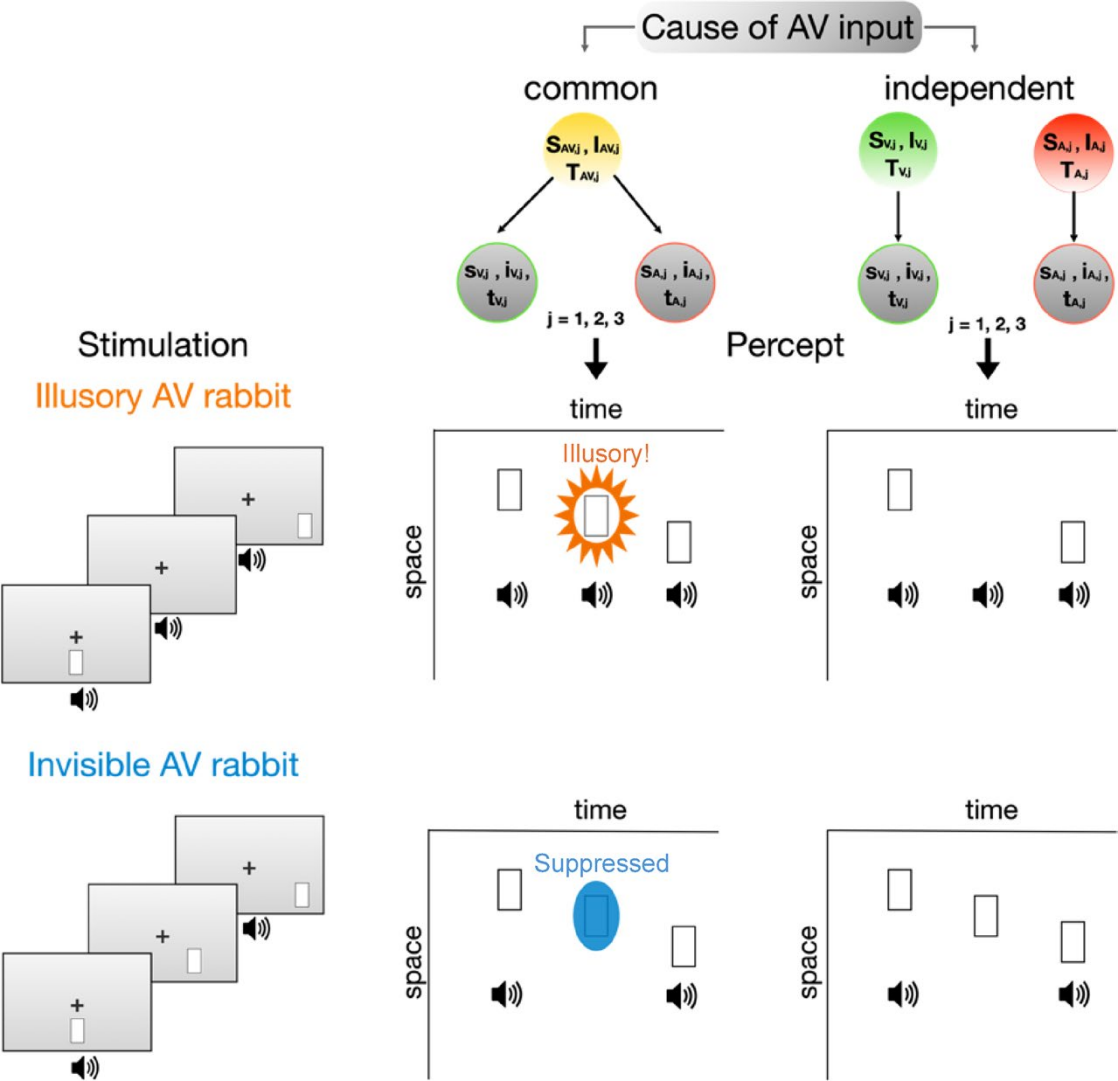
Audiovisual stimuli sequences in the Illusory and Invisible AV rabbit and perceptual outcome predictions of the generative BCI model. The figure illustrates an experimental trial in which the flash sequence moved from the center to the right. Participants were instructed to ignore the auditory stimuli and to report each perceived flash and its location. The Bayesian framework (top) posits that observers infer whether the auditory and visual inputs originated from two potential causal structures: common cause or independent causes. Observers combine causal priors with accumulating sensory evidence across time (t), space (s) and intensity (i) cues for the three different stimulus pairs (A, V, or AV, j = 1,2,3) to infer causality. If observers infer a common cause, they integrate the AV cues, leading to Illusory and Invisible AV Rabbit Illusions. In the Illusory AV Rabbit (left, middle), a flash-beep pair is followed by a single beep, and then a second flash-beep pair. When observers infer a common cause of the AV inputs, this sequence of stimuli can result in an illusory flash that is localized in between the first and the second flashes (middle of middle panel, highlighted in orange). This indicates postdictive crossmodal processing, wherein the last flash-beep pair retroactively influences the perceived location of the illusory flash. In the Invisible AV Rabbit (left, bottom) a flash-beep pair is followed by a single flash, and then a second flash-beep pair. If observers infer a common cause of the AV inputs, this sequence of stimuli can result in an invisible flash, i.e., a flash that is perceptually suppressed (middle of bottom panel, highlighted in blue). If observers infer independent causes, i.e., when auditory and visual inputs are processed separately (right panel), then the veridical number of flashes is perceived without postdictive influence and illusions. Importantly, both illusions indicate crossmodal postdiction, because the illusory and the suppressed flashes are reported between the first and the last veridical flash-beep pairs.

Imagine that you are walking down the street when you see a friend waving and yelling to get your attention from a distance. As you notice your friend, you may realize that you had been hearing their calls for some time but were not aware of them. In addition to the current calls, you then may become aware of the previously neglected calls. This example suggests that the processing of current information can retroactively influence the processing and perception of past stimuli. Since the seminal work on iconic memory by Sperling^1^, it has been known that our brains integrate information in sensory memories over a few hundred milliseconds. Thus, later sensory information can modify the perception of an earlier event, a phenomenon often referred to as postdiction^2,3^. By contrast, other retroactive influences suppress or erase a previous stimulus, as in the case of backward masking^3,4^. So far, the computations underlying postdiction have been primarily investigated in unisensory processing^5^. In everyday life, however, stimuli typically originate from different sensory modalities^6,7^. In such a context, a major challenge for the brain is to determine whether to integrate or segregate information from different modalities to achieve a coherent multisensory percept^8^. Specifically, the brain must infer causality between the different sensory channels so that sensory information arising from a common cause can be integrated across modalities, with the weights proportional to the relative sensory precision, as expressed in the Bayesian Causal Inference (BCI) framework^9–11^ (Fig. 1). Similarly, different sensory modality stimuli should be segregated if they have independent causes. In this causal inference framework, internal prior expectations are combined with the likelihoods, i.e.,the incoming sensory evidence from the external stimuli, to infer causality and perceive latent properties of the environment according to their causal structure. While the BCI model accounts well for non-postdictive multisensory processing^12^, it remains unclear whether it can also explain the computational mechanisms underlying crossmodal postdiction.

Previous studies using the BCI framework have improved our understanding of the computations underlying multisensory integration (^13–15^. For example, the Sound-Induced Flash Illusion, wherein a single flash that is presented alongside two rapid tones, is perceived as two flashes, has been found to involve causal inference^16,17^. A modified version of this illusion includes postdictive crossmodal effects: the Illusory AV Rabbit^18^. In the Illusory AV Rabbit, flash-beep pairs are presented shortly before and shortly after a single beep or a single flash. When observers perceive the Illusory AV Rabbit Illusion, an illusory single flash appears to be spatially localized between the first and the last flashes (Fig. 1, *middle panel*). In contrast, in the so-called Invisible AV Rabbit Illusion, a flash is perceptually suppressed (Fig. 1, *bottom panel*). The BCI framework^10,12^ offers a promising approach to account for postdictive crossmodal perception^2,19,20^. To determine whether flash-beep sequences in the Illusory AV rabbit Illusion arise from a common cause or independent causes, observers integrate causal evidence for each causal structure from all flash-beeps over time, space and signal presence. Flash-beep stimuli that fall within the temporal window of multisensory integration^20,21^ increase the probability of a common cause. Specifically, the last flash-beep pair in the Illusory AV rabbit Illusion provides additional sensory evidence to inform the causal probabilities. After accumulation of causal evidence, the brain subsequently integrates (or segregates) spatiotemporal information of the preceding stimuli: in the case of a common cause, the flash-beeps are integrated with a stronger weight on the more salient auditory beeps^22^. For the middle stimulus, integration of the single beep without a flash, leads to the perception of an illusory flash in the Illusory AV Rabbit because the brain infers an additional middle flash-beep event. In the Invisible AV Rabbit, integration of a single flash without a beep leads to the perceived absence of the flash because the brain infers that the middle flash-beep event did not occur. Since causal inferences are informed also by causal evidence from the last AV event, postdictive effects arise from the last flash-beep on the middle event. Hence, if the last flash-beep increases the probability of a common cause, it should also postdictively increase the probability that the middle event is integrated. Taken together, if causal inference could explain the Illusory and Invisible AV Rabbit Illusions, we would expect that the BCI model captures postdictive crossmodal processes better than alternative non-causal models.

In this study, we presented 32 observers with an extensive set of temporally synchronous or asynchronous Illusory and Invisible AV Rabbit stimuli, as well as stimuli with changing directionality. To examine whether causal inference accounts for crossmodal postdiction in the Illusory and Invisible AV Rabbit paradigms, we developed and fitted a BCI model to estimate the causal structure underlying the flash-beep sequences, which included postdictive information from the last flash-beep event. Furthermore, we compared the BCI model with a non-causal forced-fusion (FF) model and forced-segregation (FS) models. The FF and FS models assume that the flash-beep stimuli are integrated and segregated in a mandatory fashion without accounting for the stimuli’s causal structure. To test how the last flash-beep pair postdictively influences causal inference as an explanation for the Illusory and Invisible AV Rabbit Illusions, we also compared the BCI model with a non-postdictive BCI model (BCI-NP) that ignored causal information from the last flash-beep event. This was a critical comparison to test how the BCI model implicitly explains crossmodal postdiction.

## Results

To investigate the computational mechanisms underlying Illusory and Invisible AV Rabbit Illusions, we presented participants with flash-beep sequences and asked them to report each perceived flash and its location. Compared to the original setup^18^, we added experimental conditions (See Methods) to enable model fitting and to further improve the validity of the paradigm. To reduce a behavioral response bias that might occur if the flash sequence always moved in one direction, e.g. continuously from left to right, we added conditions in which the flash sequence changed its direction (e.g., the first flash was presented in the center, the second flash to the right, and the third flash to the left). Due to the presence of direction-changing conditions, the location of the last flash could not directly be predicted based on the location of the first or second flash. This made the direction of the stimuli as unpredictable as possible, which further reduced response bias^23^. Furthermore, conditions with a temporal asynchrony between visual and auditory inputs were added to test the range of the temporal binding window for the Illusory and Invisible AV Rabbit Illusions. Finally, we included multisensory (audiovisual) and unisensory (visual only) control conditions to improve the parameter estimations in the computational models.

We quantified the strength of the illusions as illusion rates, that is the proportion of trials in which a middle flash was perceived (Illusory AV Rabbit) even though it was absent, or a veridical middle flash was not perceived (Invisible AV Rabbit). Further, responses were only counted as illusions if the participants located the flashes on specific locations and in a certain order. For instance, for the illusory rabbit, a trial response was considered as an illusion only if the participant correctly located and reported flashes in the first and third position, as well as an extra illusory flash in the middle in the corresponding respective order.

To first test whether the direction of the flash sequence influenced illusion rates, following normality checks, Wilcoxon signed-rank tests or paired t-tests were implemented to compare illusion rates between the right and left motion conditions. Following Bonferroni correction (corrected *p*-value = 0.005, for 10 pairwise comparisons), there were no significant differences between left and right motion directions. Therefore, we combined left and right motion trials in the following analysis steps.

### Illusion rates are higher in illusion conditions compared to control conditions

**Fig. 2:**
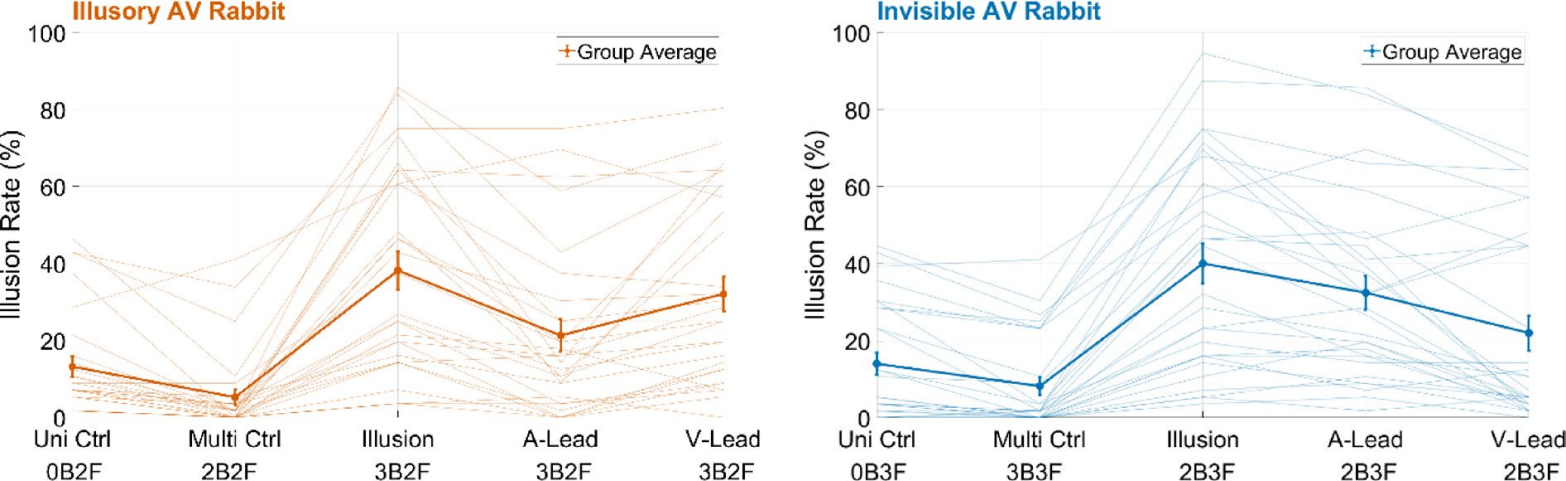
Illusion rates for the Illusory and Invisible AV Rabbits are higher in synchronous conditions than in the control and asynchronous conditions. The figure shows illusion rates for the unisensory and multisensory control conditions for the Invisible (right, blue) and the Illusory (left, orange) AV Rabbits. The number of flashes (F) and beeps (B) for each condition is demonstrated under the corresponding label (for instance, 0 beeps and 2 flashes for Illusory AV Rabbit unisensory control condition, 02BF). Thicker opaque lines show the average across subjects and thinner translucent lines show individual data. The average illusion rates for the synchronous illusion conditions were around 40%, which were higher than the illusion rates in the multisensory and unisensory control conditions. (Uni Ctrl = unisensory control; Multi Ctrl = multisensory control; A-Lead = auditory-lead asynchronous condition; V-Lead = visual-lead asynchronous condition.)

In line with the original study^18^, we compared the illusion rates for the two illusion conditions with unisensory and multisensory control conditions (Fig. 2). Both illusion conditions showed significantly higher illusion rates compared to the unisensory and multisensory control conditions (Illusory AV Rabbit: against unisensory control,

*p* = 3.75 × 10^−6^, *z* = 4.62, against multisensory control, *p* = 3.76 × 10^−6^, *z* = 4.62; Invisible AV Rabbit: against unisensory control, *p* = 1.03 × 10^−5^, *z* = 4.41; against multisensory control, *p* = 3.76 × 10^−6^, *z* = 4.62). Furthermore, the illusion rates were significantly higher for the unisensory compared to the multisensory control conditions for both illusions (Illusory AV Rabbit: *p* = 7.49 × 10^−5^, *z* = 3.96; Invisible AV Rabbit: *p* = 1.40 × 10^−4^, *z* = 3.81). These findings largely replicate the results of Stiles et al.^18^.

### Audiovisual temporal asynchrony is associated with lower illusion rates

Next, we examined the effect of stimulus asynchrony between visual and auditory stimuli. We implemented two types of temporal asynchronies: (i) an auditory lead, in which the auditory stimulus sequence started 225 ms before the visual stimulus sequence, resulting in a 225 ms stimulus onset asynchrony the first auditory beep and the first visual flash (Fig. 4), and (ii) a visual lead condition, in which the visual stimulus sequence started 225 ms before the auditory stimulus sequence. Nonparametric tests comparing the synchronous and asynchronous conditions revealed higher illusion rates for the synchronous conditions for both for the illusory AV rabbit (against A-Lead Asynchrony,

*p* = 3.63 × 10^−5^, *z* = 4.13, against V-Lead Asynchrony, *p* = 0.01, *z* = 2.51) and the invisible AV rabbit (against A-Lead Asynchrony, *p* = 4.2 × 10^−3^, *z* = 2.86, against V-Lead Asynchrony, *p* = 1.21 × 10^−5^, *z* = 4.38). Thus, temporal asynchrony reduces both illusions, which is probably the case because the sequences of auditory and visual stimuli fall outside the AV temporal integration window in asynchronous conditions.

### BCI model outperforms the forced-fusion, forced-segregation and non-postdictive models

**Fig. 3:**
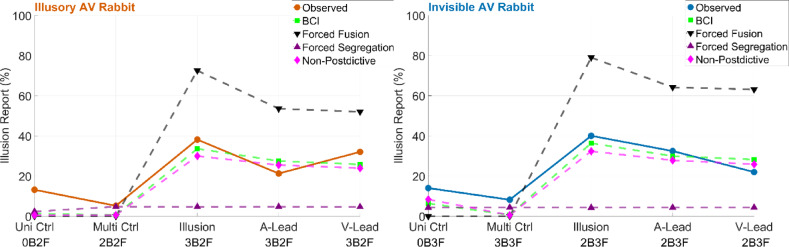
Model predictions demonstrate the BCI model’s superiority over three competing models in explaining the Invisible and Illusory AV Rabbit Illusions. The group-averaged observed data (orange in the left and blue in the right panel) and the predicted illusion rates from the fitted BCI (green), forced-fusion (FF, black), forced-segregation (FS, purple), and non-postdictive BCI (BCI-NP, pink) models are shown for the Invisible (right) and Illusory (left) AV Rabbits. Predicted illusion rates are shown for unisensory and multisensory control conditions, the illusion condition, and for asynchronous AV conditions. The BCI model shows the best overall data fit for both illusions, as demonstrated by Bayesian model comparison (Table 1). Uni Ctrl = unisensory control; Multi Ctrl = multisensory control; A-Lead = auditory lead asynchronous condition; V-Lead = visual lead asynchronous condition.

We fitted four computational models (BCI, FF, FS and BCI-NP) to each participant’s data. The main difference between the BCI, FF and FS models was the causal prior. Only the BCI model among the BCI, FF and FS estimated the stimuli’s causal structure from a causal prior and sensory evidence. In contrast, the FF and FS models assumed either a common cause leading to forced integration (i.e. equivalent to a causal prior fixed to 1) or independent causes leading to forced segregation (i.e. a causal prior fixed to 0), respectively. Next, we investigated how the sensory evidence of the third flash-beep pair was used postdictively in the BCI model to calculate posterior causal probabilities. To accomplish this, we excluded the posterior causal probability of the third flash-beep pair from the combination of posterior probabilities across all flash-beep pairs. Using this extended non-postdictive BCI model, we computed predictions about the flash reports to determine if sensory evidence from the last beep-flash pair modulates the illusory rates. Interestingly, the non-postdictive BCI (BCI-NP) model underestimated the rates of the AV rabbit illusion (Fig. 3). Finally, we computed a simulation in which we increased the spatial uncertainty of the BCI model to investigate the role of spatial auditory and visual precision on both illusions. Notably, this simulation significantly underestimated the illusion rates (Supplementary Fig. 2), demonstrating that both illusions strongly rely on spatial processing. This differs from the classical Sound-Induced Flash Illusion paradigms, in which the illusory flash is induced without a postdictive change in its location.

In the Illusory AV Rabbit, the BCI model explains the location, timing and presence of the illusory flash from causal inference: If a common cause of the flash-beep sequence is inferred, the model integrates the spatiotemporal and intensity estimate of the flashes and beeps proportional to their precision. Since the auditory cues are salient and originate from the center, the model predicts the second flash-beep at the middle location and at an intermediate timepoint. In the Invisible AV Rabbit, if the subthreshold intensity of the beep is integrated with the suprathreshold intensity of the flash, the model predicts a subthreshold second flash. This effectively suppresses the visual percept, leading to the absence of a perceived flash at the second position.

Model comparisons revealed that the BCI model outperformed the non-causal FF and FS models, including the BCI-NP model, with respect to lower average Bayesian Information Criterion (BIC) and Akaike Information Criterion (AIC) values, as well as higher R^2^ values (Table 1). Bayesian model selection revealed that the BCI model best explained the group data, with a protected exceedance probability of 0.96. FF and FS had negligible probabilities, and BCI-NP model had a probability of 0.04. Even though the BCI and BCI-NP fitted the overall data structure similarly well, the BCI model slightly outperformed the BCI-NP model in the conditions which are critical to demonstrate postdiction: Visual inspection of the BCI model’s predictions for the AV rabbit illusion showed that the model could accurately reproduce the invisible and the illusory AV rabbits and perception in the control conditions, as well as the effect of AV asynchrony (Fig. 3). In contrast, the competing models overestimated (FF model) or underestimated (FS and BCI-NP models) the illusion rates.

**Table 1:**
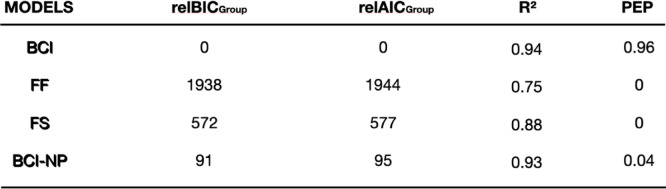
Model comparison metrics for the BCI, FF, FS and BCI-NP models show superiority of the postdictive Bayesian causal inference (BCI) compared to a forced-fusion (FF), forced-segregation (FS), and non-postdictive BCI model.

Bayesian information criterion (BIC) and Akaike information criterion (AIC) quantify the trade-off between model fit (i.e., good explanation of the data) and model complexity (i.e., a sparse number of parameters). At group level, participant-specific relBIC_Group_ and relAIC_Group_ are summed over all participants (n.b. smaller relBIC_Group_ and relAIC_Group _values indicate that a model provides a better explanation of our data). Nagelkerke’s coefficient of determination (R^2^) measured the proportion of explained variance against a null model of random guesses across 6 response options (i.e., across-participant mean reported). In a random-effects Bayesian model comparison, the protected exceedance probability (PEP) quantifies the probability that one model is more prevalent than competing models at group level, beyond which model frequencies could arise by random variations.

## Discussion

In this study, we examined the computational mechanisms underlying crossmodal perception in the Audiovisual Rabbit Illusion. Our analysis revealed three main findings. First, we observed higher illusion rates in the two illusion conditions (Illusory and Invisible AV Rabbit) compared to unisensory and multisensory control conditions, even when experimentally controlling for potential response biases. Second, we found that temporal asynchrony between auditory and visual inputs diminished the illusion rates of both illusions. Third, we showed that the BCI model is more accurate than other non-causal models at predicting the two illusions.

Our finding of higher illusion rates in the two illusion conditions compared to the unisensory and multisensory control conditions replicates the results of the original report by Stiles et al.^18^. This suggests that the Illusory and Invisible AV Rabbits involve postdictive crossmodal processing. In the current study, we thoroughly controlled for a possible behavioral response bias, which could have confounded the results of previous experiments. To achieve this, the first flash was always located in the center of the screen. This prevented spatial predictions about the upcoming flash sequence. In addition, we included trials in which the flash sequence changed its direction. These modifications should have significantly reduced the influence of sequence predictions and any possible resulting response bias. Thus, we consider it unlikely that response bias substantially influenced our results. Furthermore, we used predefined locations, indicated by button presses for five possible locations, to report the location of the perceived flashes, instead of selecting continuous points on the screen with a mouse. However, given the thorough measures that we took to avoid a response bias, we consider it unlikely that using predefined locations to indicate the flash locations has affected our findings, which were comparable to the original reports. Taken together, we propose that a behavioral response bias is unlikely to account for the postdictive crossmodal effects observed in the Illusory and Invisible AV Rabbits.

To investigate how temporal asynchrony influences the two illusions, we presented conditions in which the visual and auditory stimulation sequences were asynchronous by 225 ms. Compared to synchronous trials, asynchronous trials resulted in fewer illusion reports for both illusions. Thus, postdictive crossmodal effects are stronger when the respective sensory inputs are temporally aligned and weaker when they fall within the periphery of the temporal window of multisensory integration, which can last up to several hundred milliseconds^24,25^. Notably, preliminary data from Stiles et al.^2,26^ suggest that the AV rabbit illusion can be found even when the final flash-beep pair is delayed by up to 500 ms. However, the illusion rates are substantially reduced in this case. The current results, in which asynchronies of 225 ms between the sensory stimulation sequences were used, support this finding. Interestingly, we observed greater inter-subject variability among the study participants in the asynchronous conditions compared to the synchronous conditions. Furthermore, the effect of AV asynchrony on illusion rates was not symmetric for auditory compared with visual lead, and the effect was also different for the Illusory AV Rabbit compared to the Invisible AV Rabbit (Fig. 2). This might be related to the observation that asynchronies are handled differentially in the two illusions^27^. Notably, this may have presented a limitation of the current modelling approach, in which all data from both illusions were included simultaneously. In other words, our global model may not be able to determine how the two illusions handle asynchronies differently, leading to suboptimal model fitting of the asynchrony conditions. In the future, a more distinct approach combined with a deeper understanding of the mechanisms underlying the differential effects of asynchrony on the Illusory and Invisible AV Rabbits could be implemented^28–30^. Taken together, our results and the preliminary data by Stiles et al.^2,26^ demonstrate that a temporal window of multisensory integration exists, lasting a few hundred milliseconds. During this window stimuli can retroactively influence the processing and perception of preceding stimuli, even if these stimuli occur in different sensory modalities.

Consistent with studies on non-postdictive multisensory processing^14,15,31,32^, we intially fitted three models to the data: forced fusion, forced segregation, and BCI. Across the experimental conditions, the BCI model outperformed the other two models. Our results align with those of studies on the sound-induced flash illusion^17^, which has a design similar to that of the AV rabbit illusion but does not explicitly incorporate crossmodal postdiction. Shams et al.^16^ showed that the sound-induced flash illusion can be adequately explained by a BCI framework that posits an observer who optimally combines auditory and visual inputs. Our results extend this finding by demonstrating that causal inference can account for crossmodal postdiction as well.

The Bayesian framework has previously been applied to elucidate the computations underlying postdiction in unisensory paradigms, such as the flash-lag effect^33^ and the cutaneous rabbit illusion^5^. In these paradigms, a unisensory low-speed prior has been found to play an important role, contributing to both prediction and postdiction. In the current study, we did not model the perception of the audiovisual sequences using speed priors and likelihoods, but rather modeled independent auditory and visual inputs that are integrated if an observer infers a common cause. Specifically, we propose that inferring a common cause of audiovisual stimuli can generate illusory percepts retrospectively in the illusory and invisible AV rabbit paradigm. The brain’s perception of the causal structure of audiovisual events is determined by computing the spatiotemporal and numerical disparity of these events. Thus, the decision to integrate audiovisual information occurs only after the entire stimulus sequence has been presented, paving the way for crossmodal postdiction. More specifically, causal inferences are informed by sensory causal evidence across all flash-beep pairs, including the last flash-beep pair. Thus, postdictive effects of the last flash-beep on the middle event will occur through causal inference: if the last flash-beep increases the likelihood of a common cause, it retroactively increases the likelihood that the observer will infer an audiovisual middle event, leading to the AV rabbit illusions. This is because the beeps and flashes are integrated by weighing their relative sensory precision in case of a common cause inference. Due to the highly salient and precise auditory stimuli in the current paradigm, the auditory information can induce the illusory perception of a flash, which is also the case in the sound-induced flash illusion^17,34,35^.

Critically, our model accumulates sensory causal evidence across all audiovisual events: Not only the last flash-beep pair but also the first audiovisual event provides sensory causal evidence. Thus, the AV rabbit illusion also predictively depends on the first flash-beep. In other words, prediction and postdiction emerged naturally from our BCI model. To disentangle postdictive from predictive effects, we fitted another BCI model in which we reduced the postdictive component of the causal inference process, i.e., by eliminating the sensory causal evidence from the last flash-beep pair. Interestingly, the non-postdictive model underestimated the illusion reports, and it was inferior to the full BCI model. This suggests that the illusion decisively involves crossmodal postdictive interactions via causal inferences related to the processing of the last flash-beep pair. However, the non-postdictive model captured at least parts of the AV rabbit illusions, suggesting that non-postdictive processes, such as prediction from the first audiovisual event, also contribute to the illusions. In summary, our data show that the illusory and invisible AV rabbit illusions can be well described in terms of the BCI model. Similar to what has been previously shown for non-postdictive processing of multisensory stimuli^13–15,31,36^ and for postdictive unisensory processing^5,37^, our study provides evidence that crossmodal postdictive processing can be effectively accommodated by the BCI model.

Another interesting finding was that both the BCI and BCI-NP models showed a strong quantitative fit to the behavioral data, with R^2^ values of 0.94 and 0.93, respectively. Despite this similarity in fit qualities, the BCI model accounted for postdiction substantially better than the BCI-NP model, as demonstrated by a much higher protected exceedance probability (BCI = 0.96 vs. BCI-NP = 0.04). Given that the two models shared the same number of free parameters, the divergence is not simply driven by model complexity but rather in the systematic differences in each model’s ability to explain postdictive effects. Specifically, the full BCI model can integrate information across three flash-beep pairs, which allows it to collect later audiovisual evidence that retrospectively modulates the perception of preceding visual input. In contrast, the BCI-NP model’s causal inferences are not informed by the last flash-beep pair. This yields a modest, albeit consistent likelihood advantage of the BCI model that led to higher exceedance probabilities, even when the overall fit indices are comparable. Overall, these outcomes suggest a postdictive causal-inference mechanism, in which perceptual estimates are updated due to causal inferences from subsequent sensory information.

An open question concerns the neural mechanisms underlying causal inference and crossmodal postdictive processing. According to a hierarchical model of causal inference in the brain^14,15,38^ and in line with predictive coding^39^, prefrontal cortical areas may accumulate sensory causal evidence across the audiovisual sequence via recurrent message passing across the cortical hierarchy to make a causal decision^15,31,38^. Accordingly, posterior multisensory regions integrate or segregate the audiovisual representations to compute audiovisual estimates that account for the most likely causal structure. Thus, the postdictive effect of the last audiovisual event may manifest as an accumulation process of sensory causal evidence. This would be followed by a final perceptual estimate that may include an illusory or invisible flash, if the probability of a common cause is high. Recently, Stiles et al. proposed a model of re-entry postdictive processing in the visual cortex for the AV rabbit illusion^2^. In this model, the processing of the illusory flash, generated by the first flash-beep pair and the subsequent beep, is maintained long enough in the visual cortex to overlap with the processing of the second flash-beep pair. This reallocates the illusory flash between the first and the third flashes. It is also possible that such re-entry processing relies on multi-timescale oscillatory brain dynamics, including feed-forward and feedback processing between sensory and higher-order association cortices^7^. Finally, ongoing neural oscillations, particularly in the alpha band (8–12 Hz), may influence the integrative processing of auditory and visual stimulus sequences. Prestimulus alpha oscillations have been associated with crossmodal integration^40–42^ and postdiction in unisensory processing^43^. Taken together, the results of the current study allow us to draw concrete assumptions about the neural dynamics underlying the Illusory and Invisible AV Rabbit. Future studies could test these assumptions using functional neuroimaging or electrophysiological methods.

In summary, previous studies demonstrated the effectiveness of the BCI framework in elucidating the computations underlying non-postdictive multisensory integration and postdiction in unisensory paradigms. Here, we applied computational modeling to the illusory AV rabbit paradigm, which combines these two areas of research, i.e. multisensory integration and postdiction. Our results show that the BCI framework accurately accounts for crossmodal postdiction and outperforms non-causal models and non-postdictive simulation extensions. Our study provides strong evidence that the BCI framework can be applied to the processing of audiovisual stimulus sequences across the entire temporal window of multisensory integration, including crossmodal postdiction phenomena.

## Methods

### Participants

The study was approved by the Ethical Committee of the Charité – Universitätsmedizin Berlin (approval number: EA2/039/19). All methods were performed in accordance with the relevant guidelines and regulations, including the Declaration of Helsinki. Thirty-two healthy adult volunteers (15 females, 16 males, and one non-binary individual; mean age: 27 years, range: 20–41 years; one left-handed) participated in the study after providing written informed consent. Two participants were excluded from the analysis because they did not follow the instructions and could not complete the entire experimental session. After the exclusion of these participants, two more participants were excluded as their illusion rates in the unisensory and multisensory control conditions were three standard deviations higher than the group average. Data from 28 participants (13 females, 14 males and one non-binary individual; mean age: 27y, range: 20-33 years; one left-handed) were included in the final data analysis. Handedness was assessed using the Edinburgh Handedness Inventory. None of the participants reported a history of neurological or psychiatric disorders and all had normal or corrected-to-normal vision and hearing.

### Setup and stimuli

The experiment consisted of 3 training and 14 experimental blocks, which included visual-only and audiovisual stimuli. Prior to the experimental blocks, one additional block with auditory-only stimuli was presented to estimate the individual auditory precision. In this block participants had to report the number of perceived auditory beeps. Each of the 14 experimental blocks lasted around 5 min and between blocks, participants were allowed to take short breaks. At the beginning of the experiment, which had an overall runtime of about 2 h, participants were provided with verbal and written instructions regarding the task. The study was conducted in a soundproof chamber, which was dimly lit. The presentation of auditory and visual stimuli was controlled using Psychtoolbox 3.09^44^. Visual stimuli (rectangle bars) were presented on VPixx’s VIEWPixx high quality 120 Hz calibrated research-grade screen. The flashes could appear at five locations, aligned horizontally at 4° below a central fixation cross. The locations were (from left to right): -5.68°, -2.84°, 0°, 2.84°, 5.68° visual angles. Flashes (of luminance 54.1 cd/m^2^) were presented against a gray background (of luminance 8.7 cd/m^2^) with a width of 0.56° visual angle and a height of 2.4° visual angle. Each flash was presented for about 17 ms. Auditory stimuli (beeps) were presented through a loudspeaker located centrally below the display. They consisted of a square wave tone with a carrier frequency of 800 Hz and a sound pressure level of 88 dB. Each beep lasted 7 ms.

### Experimental paradigm

The Illusory and Invisible AV Rabbit Illusion paradigms were adapted from Stiles et al.^18^. To allow for the modeling of postdictive crossmodal perception, we extended the original study by various stimulation conditions (Table 2). The participants’ task was to ignore auditory stimuli and to report each perceived flash and its location in each trial by successive button presses (after each trial) on a keyboard with five response keys. The response keys on the ergonomically distributed keyboard represented the five horizontal locations where a flash could appear on the screen. To ensure that participants kept their gaze on the central fixation cross, catch trials in which the cross turned into a circle (for 100 ms, 150 ms after the beginning of a trial) were presented in 6.66% of all trials. The group average performance for the fixation catch trials was 98.7% ± 0.56 (Standard error of the mean). Participants were instructed to press the middle key when this happened. Participants were also informed that most trials contain 2 or 3 flashes, that the first flash would always be presented at the central location, and that the sequence of flashes could move in one direction (left or right) or could change direction.

**Table 2:**
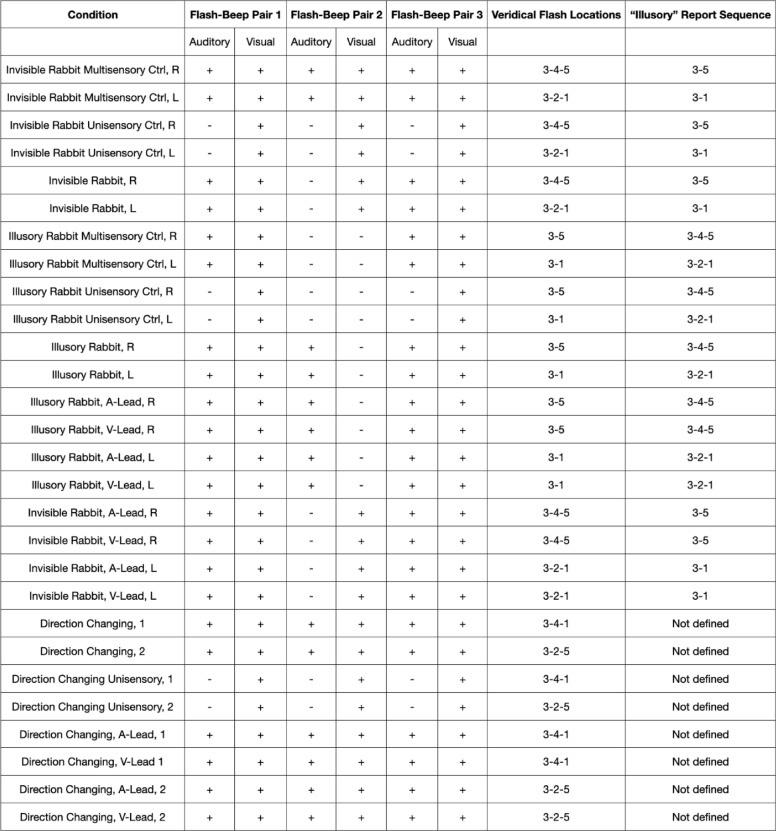
Overview of visual and audiovisual experimental conditions.

**Fig. 4:**
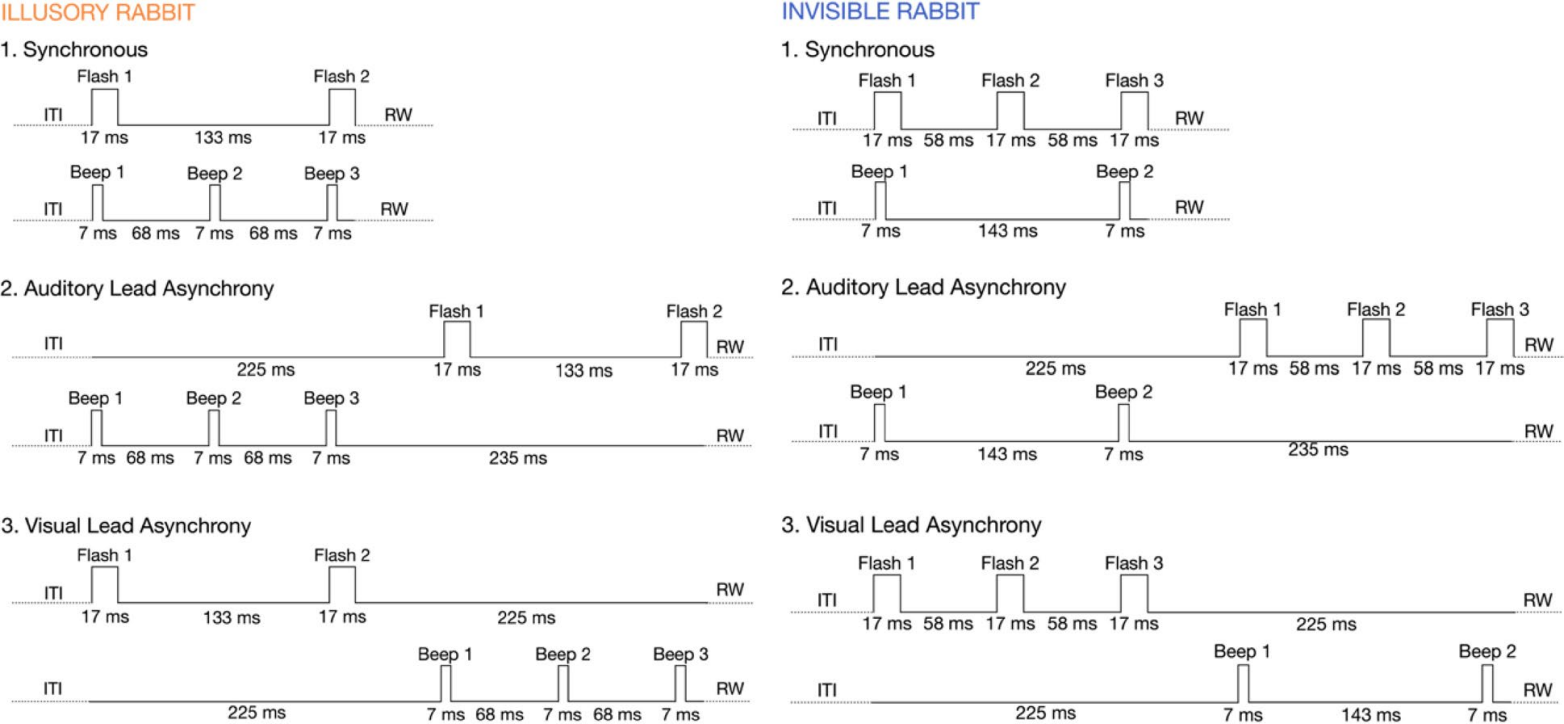
Timing of auditory and visual inputs in illusory and invisible AV rabbit trials. The figure illustrates the input timing for the synchronous AV trials (top), auditory lead AV trials (middle), visual lead trials (bottom) for the invisible AV rabbit (upper) and the illusory AV rabbit (lower). ITI = intertrial interval; RW = response window.

Stimuli are categorized for the 28 experimental conditions, from left to right, according to the number of flashes and beeps, the locations of the flashes on the screen, and the postdictive illusory reports taken into account for the behavioral analysis. For a trial report to be regarded postdictive, locations and order are essential, in addition to the number of flashes reported. For the conditions, R and L represent the right and left sequences, respectively. The “1–5” represent the locations on the screen with position 3 in the middle, 4 and 5 to the right and 1 and 2 to the left. A-Lead represents the auditory lead and V-Lead represents the visual-lead asynchrony conditions. Type 1 and type 2 are two different combinations of direction-changing conditions. 8 of the 18 conditions with a second flash presented the flash with a change of direction. Thus, the regularity of the sequence was only probabilistically predictable from the second flash. This reduced a potential explicit response bias for the illusory AV rabbit which could have occurred with only continuous center-to-periphery sequences: if a second (illusory) flash had been seen, it could have occurred on the left (2.) or right (4.) position. Twelve of the 28 conditions were asynchronous. In synchronous trials, the 7 ms auditory beeps and the 17 ms visual flashes for a given pair of flash-beeps were presented simultaneously and their stimulus onset was 75 ms apart from the following pair of flash-beeps. However, in two types of asynchronies, either auditory beeps or visual flashes were presented first, referred to here as auditory lead and visual lead, respectively (Fig. 4). Thus, only three inputs of a single modality were presented first, with a stimulus onset of 75 ms between them, and after 58 ms the sequence of the other modality was presented.

Pilot data have shown that these instructions reduce the response bias that some subjects showed without these instructions. In the experimental runs stimuli consisted of sequences of 2 or 3 flashes, which were presented with stimulus onset asynchronies of 75 ms (Fig. 4). Flashes were presented together with 0, 2 or 3 beeps that were not task relevant. In total, there were 28 experimental conditions (Table 2) and each participant completed 28 trials per condition. In each trial participants had a response window of 3 s. The inter-stimulus interval between trials varied randomly between 1.2 and 1.8 s (mean = 1.5 s). Hence, each trial lasted 4 to 5 s. In each block, participants completed 60 trials, including four catch trials and the order of the 28 experimental conditions was randomized across blocks.

### Data analysis

Data analysis was performed, and plots were generated using MATLAB version R2024a^45^. Participants’ flash reports were analyzed by computing AV rabbit illusion rates. A trial response was regarded as an illusion for the illusory AV rabbit if participants reported three consecutive flashes in the first, second and third locations (i.e., 3–4-5 or 3–2-1). For the invisible AV rabbit, a trial response was considered an illusion if participants reported two consecutive flashes in the first and third locations (i.e., 3–5 or 3–1). Illusory reports for each experimental condition are defined in Table 2. In the control trials, a “match/hit” was defined as correctly reporting the veridical order of the visual flashes on the screen. For each participant and condition, the percentage of correct responses based on illusory reports or match responses (reporting the veridical sequence) was calculated. First, Shapiro–Wilk tests were performed to investigate the normality of the data distribution. Because most of the illusion rates violated the normality assumption, the effects flash sequence direction on the illusion rates was tested with nonparametric (Wilcoxon signed rank) tests. There was no evidence for an effect of direction (left to right, or right to left). Since direction effects were not part of our research question, data from left and right directions were combined. This resulted in ten experimental conditions for the further data analysis. For each illusion condition, the main research question was whether the illusion rates differed between the illusory conditions and the unisensory and multisensory control conditions, as well as the asynchronous illusion conditions. To this end, nonparametric tests Wilcoxon signed-rank tests were computed. All *p*-values were adjusted using Bonferroni correction for multiple comparisons.

### Computational modelling of the behavioural data

To investigate whether participants adopted a causal inference or an alternative non-optimal decision strategy such as forced fusion or forced segregation, four models (BCI, non-postdictive BCI, forced-fusion and forced-segregation) were fitted to the reports of flashes and their locations. Bayesian model comparison was used to determine which model best explained participants’ behavioral data and the predictions of the causal inference and competing models for the illusion rates were examined. The BCI model combined the precision-weighted fusion estimate with the auditory segregation estimate proportional to the posterior probability of a common or independent causes, respectively. The forced fusion model^9,19^ integrated the AV stimuli, weighted by their relative precision in a mandatory fashion. Finally, the forced segregation model assumed that participants’ flash percepts were independent from any auditory inputs, i.e. beeps. Details on the BCI model and the fitting to audiovisual perception on singular dimension such as spatial location and stimulus number can be found in Koerding et al.^10^ and Rohe et al.^10,14^. For perception of multivariate cues such as our flash-beep sequences, the approach implemented by Samad et al.^46^ was extended to signals described by location, timing and intensity cues (Fig. 1). The absence of a signal was conceived as a stimulus of zero intensity.

Our generative BCI model assumed that common (C = 1) or independent (C = 2) causes were determined by sampling from a binomial distribution with the causal prior p(C = 1) = p_common_. For a common cause, each of the three independent flash-beep pairs S_AV,j_ was drawn from a common trivariate normal distribution N(μ_P_, Σ_P_), which modeled a cue for location S_AV,j_ (along the azimuth), time T_AV,j_ (relative to fixation), and intensity I_AV,j_ (which can be above or below a perceptual threshold) of the flash-beep pair. We assumed independence of the three stimulus components, so that the trivariate normal distribution was characterized by the prior spatial, temporal, and intensity means μ_S,P, j,_ μ_T,P,j, _ μ_I,P,A, j _for beeps and μ_I,P,V,j_   for flashes and the diagonal covariance matrix of the prior spatial, temporal, and intensity standard deviations σ_S,P_, σ_T, P_ and σ_I,P_ (i.e., the same for j ∈ {1, 2, 3}).

For two independent causes, the “true” distal auditory and visual locations (S_A,j_ and S_V,j_), timing (T_A,j_ and T_V,j_), and intensity (I_A,j_ and I_V,j_) of beeps and flashes were drawn independently from the prior distribution. The observer does not have direct access to the distal beep-flash sequences but needs to infer and estimate their properties from proximal noisy sensory inputs x_A,j_ = (s_A,j_, t_A,j_, i_A,j_) and x_V,j_ = (s_V,j_, t_V,j_, i_V,j_). Sensory noise was introduced by sampling the sensory inputs independently from trivariate normal distributions. These distributions were centered on the experimentally defined auditory (respectively visual) temporal and spatial location as well as intensity of the stimuli. If a stimulus was presented or absent in a sequence, the intensity was set to 1 or 0, respectively. We assumed independence of the sensory inputs’ cues so that the variance is characterized by a diagonal covariance matrix with parameters σ_sA_ , σ_tA_ , σ_iA_ (respectively σ_sv_ , σ_tV_ , σ_iV_). Further, we assumed that an observer only reported a flash if its intensity i_V,j_ was above a perceptual threshold Φ_I_. Overall, the basic generative model included the following 11 parameters: the causal prior p_common_, the prior’s spatial, temporal and intensity standard deviations σ_S,P_, σ_T,P_ and σ_I,P_, the auditory standard deviations σ_sA_ , σ_tA_ , σ_iA_, and the visual standard deviations σ_sV_ , σ_tV_ , σ_iV_, and the perceptual intensity threshold Φ_I_. Please note that we denote all variables and parameters related to the distal stimuli with uppercase letters and all variables and parameters related to the proximal sensory inputs with lowercase letters. The prior’s spatial mean μ_S,P,j_ was fixed to zero for all three flash-beep pairs j, assuming that observers expect unbiased, centered audiovisual stimuli. The prior’s temporal mean μ_T,P,j_, were fixed to 47, 105 and 163 ms for j ∈ {1, 2, 3} as experimentally defined (Fig. 4). Only for the intensity prior, we differentiated between a visual and auditory intensity prior to reflect different a priori likelihoods of stimulus occurrence which can be quickly learned by the observer across all experiment conditions: The prior’s intensity means μ_I,P,V,j_ was fixed to 1 for j ∈ {1, 3} and to 0.65 for j ∈ {2}, for the visual stimuli, whereas μ_I,P,A,j_ was fixed to 0.78 for j ∈ {1, 3} and to 0.5 for j ∈ {2}, for the auditory stimuli. These μ_I,P,V,j_ and μ_I,P,A,j_ values represent the proportion of trials across all conditions in which a stimulus was indeed presented in the experiment.

Because the observer does not have direct access to the generative process, the observer needs to infer the causal structure and perceptually estimate properties of the flash-beeps from sensory inputs: For the causal inference according to the full postdictive BCI model, we assume that the observer accumulates the causal sensory evidence across the three flash-beep events and combines the evidence with the causal prior: Given flash-beeps’ vectorial sensory inputs x_A,j_ and x_V,j_, the observer infers the posterior probability of the underlying causal structure by combining the causal prior with the sensory likelihood according to Bayes rule:

1 $$\begin{aligned} & {\mathrm{p}}({\mathrm{C}} = 1{\mid }x_{A,1} ,x_{V,1} ,x_{A,2} ,x_{V,2} ,x_{A,3} ,x_{V,3} ) \\ = & \frac{{{\mathrm{p}}(x_{A,1} ,x_{V,1} ,x_{A,2} ,x_{V,2} ,x_{A,3} ,x_{V,3} {\mid }{\mathrm{C}} = 1){\mathrm{p}}_{{{\mathrm{common}}}} }}{{{\mathrm{p}}(x_{A,1} ,x_{V,1} ,x_{A,2} ,x_{V,2} ,x_{A,3} ,x_{V,3} {\mid }{\mathrm{C}} = 1){\mathrm{p}}_{{{\mathrm{common}}}} + {\mathrm{p}}(x_{A,1} ,x_{V,1} ,x_{A,2} ,x_{V,2} ,x_{A,3} ,x_{V,3} {\mid }{\mathrm{C}} = 2){\mathrm{p}}_{{{\mathrm{common}}}} }} \\ \end{aligned}$$

The sensory likelihood under a common cause assumption (C = 1) is accumulated over three independent flash-beeps and their independent spatial, temporal and intensity cues:

2 $$p(x_{A,1} ,x_{V,1} ,x_{A,2} ,x_{V,2} ,x_{A,3} ,x_{V,3} |C = 1) = \prod\limits_{j = 1}^{3} p (s_{A,j} ,s_{V,j} |C = 1)\prod\limits_{j = 1}^{3} p (t_{A,j} ,t_{V,j} |C = 1)\prod\limits_{j = 1}^{3} p (i_{A,j} ,i_{V,j} |C = 1)$$

The sensory likelihood for each cue can be obtained by integrating over flash-beep pairs^7^, e.g. for the spatial component S_AV,j_ (and likewise for the temporal and intensity components):

3 $$p(s_{A,j} ,s_{V,j} {\mid }C = 1) = \int p (s_{A,j} {\mid }S_{AV,j} )p(s_{V,j} {\mid }S_{AV,j} )p(S_{AV,j} )dS_{AV,j}$$

The three factors in the integral are Gaussian and can therefore be solved analytically:

4 $$\begin{aligned} p(s_{A,j} ,s_{V,j} {\mid }C = 1)  = & \frac{1}{{2\pi \sqrt {\sigma _{{s_{V} }}^{2} \sigma _{{s_{A} }}^{2} + \sigma _{{s_{V} }}^{2} \sigma _{{S,P}}^{2} + \sigma _{{s_{A} }}^{2} \sigma _{{S,P}}^{2} } }} \\ \quad & \cdot \exp \left( { - \frac{1}{2} \cdot \frac{{(s_{{V,j}} - s_{{A,j}} )^{2} \sigma _{{S,P}}^{2} + (s_{{V,j}} - \mu _{{S,P,j}} )^{2} \sigma _{{s_{A} }}^{2} + (s_{{A,j}} - \mu _{{S,P,j}} )^{2} \sigma _{{s_{V} }}^{2} }}{{\sigma _{{s_{V} }}^{2} \sigma _{{s_{A} }}^{2} + \sigma _{{s_{V} }}^{2} \sigma _{{S,P}}^{2} + \sigma _{{s_{A} }}^{2} \sigma _{{S,P}}^{2} }}} \right) \\ \end{aligned}$$

In an analogous fashion to Eq. 3, we can derive and compute the sensory likelihood under an independent cause assumption (C = 2):

5 $$p(s_{A,j} ,s_{V,j} |C = 2) = \left( {\int p (s_{A,j} |S_{A,j} )p(S_{A,j} )dS_{A,j} } \right)\left( {\int p (s_{V,j} |S_{V,j} )p(S_{V,j} )dS_{V,j} } \right)$$

The Gaussian distributions allow for an analytic solution:

6 $$P(s_{A,j} ,s_{V,j} |C = 2) = \frac{1}{{2\pi \sqrt {(\sigma _{{s_{V} }}^{2} + \sigma_{S,P}^{2} )(\sigma _{{s_{A} }}^{2} + \sigma_{S,P}^{2} )} }} \cdot \exp \left( { - \frac{1}{2}\left( {\frac{{(s_{V,j} - \mu_{S,P,j} )^{2} }}{{\sigma _{{s_{V} }}^{2} + \sigma_{S,P}^{2} }} + \frac{{(s_{A,j} - \mu_{S,P,j} )^{2} }}{{\sigma _{{s_{A} }}^{2} + \sigma_{S,P}^{2} }}} \right)} \right)$$

Effectively, the posterior probability of the underlying causal structure (Eq. 1) combines the sensory likelihoods (Eqs. 4 and 6) with the causal prior: Critically, the sensory likelihoods under both causal structures are accumulated (i.e., mathematically multiplied) across the cues of all three flash-beep pairs according to Eq. 2.

For optimal perceptual inference of flash-beep estimates in the case of a common cause (C = 1), the audiovisual estimate of a spatial, temporal or intensity of the stimulus (e.g., the spatial component $\widehat{\mathrm{S}}_{\mathrm{AV,C=1,j}}$) is obtained by combining the auditory and visual cues as well as the prior weighted by their relative reliability as quantified by the inverse of sensory variances (i.e., fusion estimate):

7 $$\hat{S}_{{AV,C = 1,j }} = \frac{{\frac{{s_{V,j} }}{{\sigma _{{s_{V} }}^{2} }} + \frac{{s_{A,j}  }}{{\sigma_{{s_{A} }}^{2} }} + \frac{{\mu_{S,P,j} }}{{\sigma_{S,P}^{2} }}}}{{\frac{1}{{\sigma_{{s_{V} }}^{2} }} + \frac{1}{{\sigma_{{s_{A} }}^{2} }} + \frac{1}{{\sigma_{S,P}^{2} }}}}$$

In the case of independent causes (C = 2), the optimal estimates of the unisensory visual stimulus component (e.g., the spatial component $\widehat{\mathrm{S}}\text{V,C=2, j}$) are independent from the auditory cues (i.e. segregation estimate):

8 $$\hat{S}_{{V,C = 2,j }} = \frac{{\frac{{s_{V,j} }}{{\sigma _{{s_{V} }}^{2}}} + \frac{{\mu_{S,P,j} }}{{\sigma_{S,P}^{2} }}}}{{\frac{1}{{\sigma _{{s_{V} }}^{2} }} + \frac{1}{{\sigma_{S,P}^{2} }}}}$$

Because the observer needs to infer whether the sensory cues come from common or independent sources, the observer’s causal inference is subject to a uncertainty which also influences the observer’s perceptual inference: To account for observer’s causal uncertainty, the model computes final task-relevant spatial, temporal and intensity estimates of the distal stimuli (e.g. spatial $\widehat{\mathrm{S}}_{\mathrm{V,j}}$) by combining the fusion estimate (e.g., $\widehat{\mathrm{S}}_\mathrm{AV,C=1,j}$ for C = 1) and the task-relevant segregation estimate (e.g., $\widehat{\mathrm{S}}_\mathrm{V,C=2,j}$ for C = 2) depending on the posterior probabilities of the estimates’ underlying causal structures. In the ‘model averaging’ strategy of the BCI model, the observer weighs the estimates in proportion to the posterior probabilities of their underlying causal structures, for example for the posterior visual spatial estimate $\widehat{\mathrm{S}}_\mathrm{V,j}$:

9 $$\hat{S}_{{V,j }} = p(C = 1|x_{A,1} ,x_{V,1} ,x_{A,2} ,x_{V,2} ,x_{A,3} ,x_{V,3} ) \cdot \hat{S}_{{AV,C = 1,j }} + (1 - p(C = 1|x_{A,1} ,x_{V,1} ,x_{A,2} ,x_{V,2} ,x_{A,3} ,x_{V,3} )) \cdot \hat{S}_{{V,C = 2,j }} \,$$

Note that the observer can also combine the numerical estimates according to different decision strategies such as model selection and probability matching^47^. We compared the Bayesian ‘model averaging’ decision strategy, which take causal uncertainty into account, with two additional non-optimal heuristic strategies: First, a classical forced-fusion model^9^ that mandatorily assumes a common cause (i.e., p_common_ = 1), thus always selecting the fusion estimate (Eq. 7). Second, a forced segregation model that mandatorily assumes independent causes (i.e., p_common_ = 0), thus always selecting the segregation estimate (Eq. 8). The forced fusion and segregation models both have one free parameter less (i.e., p_common_), thus reducing the complexity of model compared to the full BCI model.

Finally, we investigated a potential postdictive mechanism from the last beep-flash pair on the perception of the illusory and invisible rabbit by testing the influence of the sensory evidence of the third flash-beep pair on causal inferences in the BCI model. In the non-postdictive BCI model, we assumed that the observer accumulates the causal evidence only across the first and second flash-beep events but ignores the third event. Thus, we excluded the sensory evidence of the third flash-beep pair from the combination of sensory likelihoods across all flash-beep pairs (i.e., Eq. 2, j ∈ {1, 2}).

To compare the four candidate models, we fitted each model to participants’ behavioral reports of the perceived number and location of flashes based on the predicted distributions of the visual estimates individually (i.e. the marginal distributions, e.g. for the location component: p($\hat{\mathrm{S}}_\text{V,j }\mathrm{|S}_\mathrm{A,j}\text{, S}_\mathrm{V,j}$)). The predicted distribution was obtained by marginalizing over the sensory inputs x_A,j_ and x_V,j_, i.e. internal variables that are not accessible to the experimenter^10^. These distributions were generated by simulating x_A,j_ and x_V,j_ in 5000 trials (i.e. continuous variables sampled from Gaussian distributions) for each experimental conditions and inferring spatial estimates $\hat{\mathrm{S}}_\mathrm{V,j}$ and intensity estimates $\hat{\mathrm{I}}_\mathrm{V,j}$ from Eqs. 1–9 (n.b.: $\hat{\mathrm{T}}_\mathrm{V,j}$ is not relevant because we did not obtain a response on the timing of the flashes). To link the predicted distributions of participants’ reported number of flashes for flash-beep events one, two and three, we assumed that an observer reported a flash if $\hat{\mathrm{I}}_\mathrm{V,j}$ was above the perceptual intensity threshold Φ_I_. To link the predicted distributions of the spatial estimates $\hat{\mathrm{S}}_\mathrm{V,j}$ of perceived flashes to participants’ visual location judgments (i.e., five possible locations), we assumed that participants selected the button that is closest to $\hat{\mathrm{S}}_\mathrm{V,j}$ and binned $\hat{\mathrm{S}}_\mathrm{V,j}$ accordingly into a five-bin histogram. If observers did not perceive a flash because the intensity estimate $\hat{\mathrm{I}}_\mathrm{V,j}$ was below threshold (e.g., the second flash in the invisible rabbit illusion), we counted these simulated trials into a sixth bin of the histogram. We computed these predicted six-bin multinomial distributions for each of the three flash-beep events of a condition, and three distributions separately for each of the experimental conditions. Using the predicted multinomial distributions, we computed the log likelihood of participants’ three flash reports in each condition and summed the log likelihoods across all three flash reports and all experimental conditions of this task.

To obtain maximum likelihood estimates for the 11 parameters of the BCI model (p_common_, σ_S,P_, σ_T,P_, σ_I,P_, σ_sA_, σ_tA_, σ_iA_, σ_sV_, σ_tV_, σ_iV_, Φ_I_), we used a Bayesian optimization algorithm as implemented in the BADS toolbox^48^ and initialized this optimization algorithm with multiple different random parameters to prevent local minima. To keep the model simple, spatial, temporal and intensity cues were treated as statistically independent and assumed to carry no specific information in their prior distributions. Consequently, the standard deviations for the spatial, temporal and intensity priors (i.e. σ_S,P_, σ_T,P_, σ_I,P_ ) were set to large values to mimic uniform distributions (i.e., non-informative priors). To improve the parameter estimation of our four candidate models, we included unisensory condition into an initial parameter estimation: First, we fitted 3 visual parameters (σ_sV_ , σ_tV_ , σ_iV_) data from six unisensory visual conditions with 50 randomized parameter initialisations, with fixing intensity threshold parameter to 0.5. Plausible lower and upper boundaries were chosen for the fits (Supplementary Table 1), taking the experimental paradigm into account. From this unisensory fit, two parameters (σ_sV_, σ_iV_), related to the cues of the visual stimuli, were fixed. The temporal visual parameter σ_tV_ was not fixed, but it was fitted in the multisensory conditions. Because there was no asynchrony in the unisensory visual conditions, fixing σ_tV_ based on unisensory visual conditions would severely underestimate the asynchrony effects in visual perception. Finally, since the participants were informed to ignore the sound for the task, and almost perfectly distinguished 2 and 3 beeps in the unisensory auditory precision block, the auditory intensity standard deviation σ_iA_ was fixed to 10^–10^. This reflected that the participants had very low uncertainty about auditory intensity and nearly perfectly detected the presence or absence of beeps (Supplementary Table 1). The remaining four parameters were then fitted to the AV conditions with 100 random parameter set initialisations, with each optimization iteration simulating 5000 trials with sensory inputs x_A,j_ and x_V,j_. We report the results (i.e., model comparisons and parameters) for models with the highest log likelihood across these initializations (Table 1 and Supplementary Table 1). To validate that the models’ parameters can be robustly and accurately estimated from the data using our fitting procedures, we performed a parameter recovery: We re-fitted the BCI model to data simulated by the model. Parameter recovery showed good reproduction of the parameters in our fitting procedure (Supplementary Fig. 3). Nevertheless, the parameter recovery was more accurate for the causal prior than for the temporal and spatial parameters. Even though the accuracy was in the range of a previous multisensory study^49^, our findings indicate that the latter parameters were less identifiable in our experimental design. Even though we manipulated the location and timing of flashes and beeps, we did not manipulate those experimental parameters as systematically as in previous BCI experiments^10,15^, which may have contributed to dependencies between the temporal and spatial auditory and visual parameters.

To identify the optimal model that explains participants’ data, we compared the four candidate models using the Bayesian Information Criterion (BIC; BIC =  − 2 × LL + m × ln(n), LL = log likelihood, *m* = number of parameters, *n* = number of data points) and Akaike Information Criterion (AIC; AIC = 2 × m − 2 × LL) as an approximation to the model evidence^50^. BIC and AIC were aggregated at the group level, i.e. participant-specific BICs and AICs were first expressed relative to the best model individually and then summed over all participants (Table 1). We performed Bayesian model comparison^51^ at the random-effects group level as implemented in SPM12^52^ to obtain the protected exceedance probability for each of the four candidate models. Protected exceedance probability quantifies the probability that a given model is more likely than any other model, beyond differences due to chance^51^. To generate predictions for participants’ flash reports based on the four candidate models, we simulated new x_A,j_ and x_V,j_ for 5000 trials for each experimental condition using the fitted model parameters of each participant. For each simulated trial, we sampled the candidate models’ response from the multinomial predicted distributions and analyzed the models’ responses exactly like participants’ behavioral responses. To reduce the influence random sampling on the models’ predictions, we simulated the tenfold number of trials for each model in each participant.

## Electronic Supplementary Material

Below is the link to the electronic supplementary material.

Supplementary Material 1

## Data Availability

The collected data and generated datasets of this study are available upon reasonable request.
