# Supplementary material for: Causal inference shapes crossmodal postdiction in multisensory integration
Source: Sci Rep. 2026 Feb 21;16:7490. doi: 10.1038/s41598-026-36884-6 (PMC12929559; doi:10.1038/s41598-026-36884-6)
Supplement: Supplementary file 1 — Supplementary Material 1 [file 41598_2026_36884_MOESM1_ESM.docx]

**SUPPLEMENTARY MATERIAL**

**Supplementary Table 1**

**
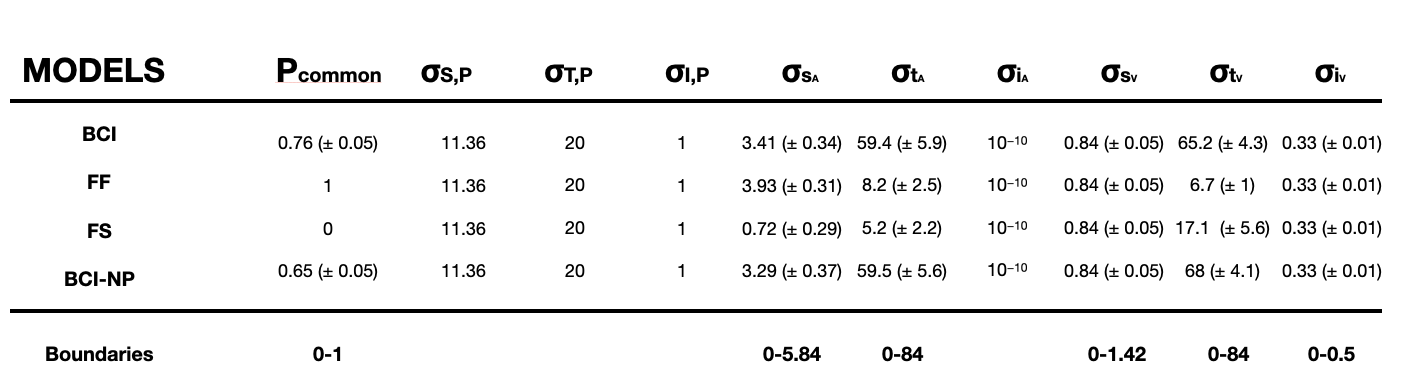
**

**Supplementary Table 1.** **Fitted parameters for the BCI, FF and FS models.** Average parameter values (+- SEM) for the fitted 4 parameters (p_common_, σ_sA_, σ_tA_ and σ_tV_) across 28 participants, as well as the fixed parameters (σ_S,P,_ σ_T,P,_ σ_I,P,_ σ_sV_ , σ_iA_ and σ_tV_) as well as the plausible upper and lower range boundaries for the BADS optimization for the parameter fitting.

**Supplementary Figure 1**


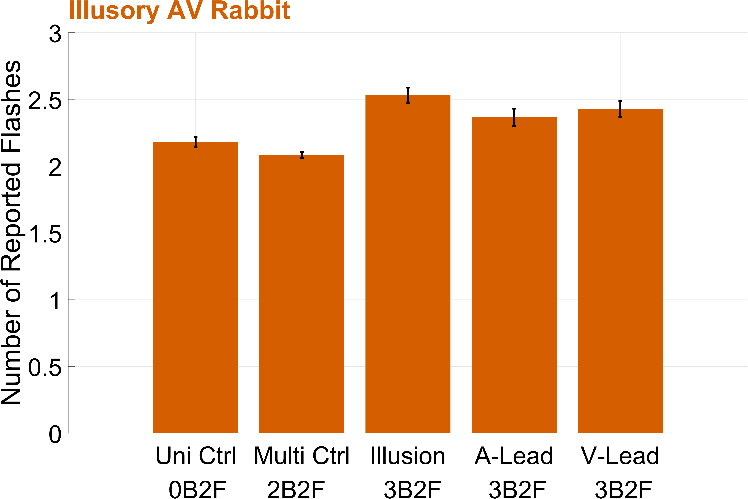

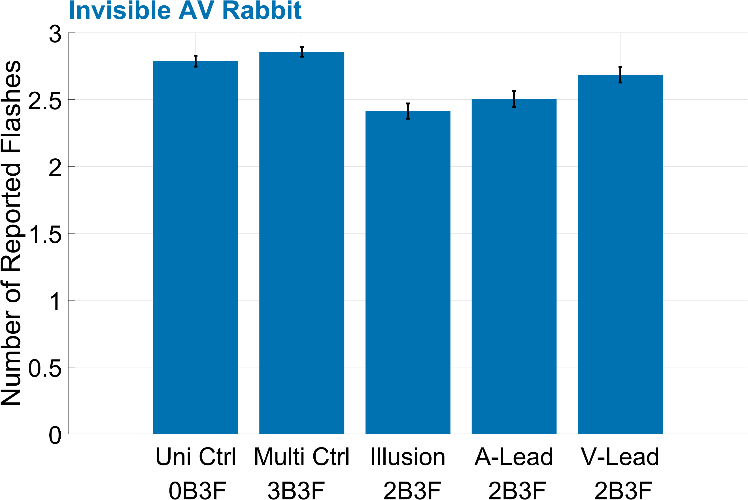


**Supplementary Figure 1. Number of flashes reported for the Illusory and Invisible AV Rabbits are higher in synchronous conditions than in the control and asynchronous conditions.** The figure shows number of flashes reported for the unisensory and multisensory control conditions for the Illusory (left, orange) and Invisible (right, blue) AV Rabbits. The number of flashes (F) and beeps (B) veridically represented for each condition is demonstrated under the corresponding label (for instance, 0 beeps and 2 flashes for Illusory AV Rabbit unisensory control condition, 02BF). (Uni Ctrl = unisensory control; Multi Ctrl = multisensory control; A-Lead = auditory-lead asynchronous condition; V-Lead = visual-lead asynchronous condition.)

**Supplementary Figure 2**

**
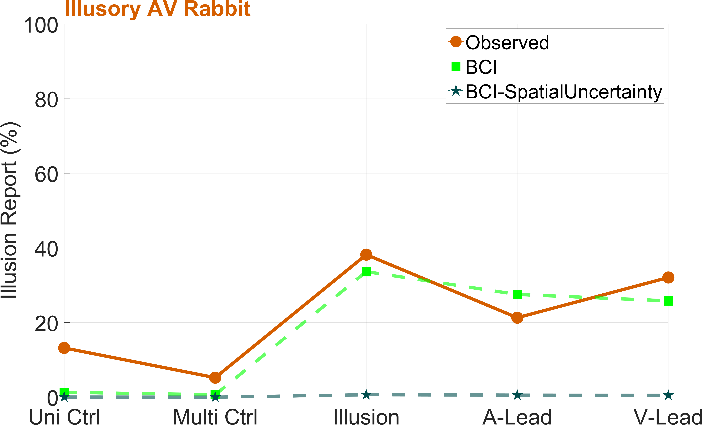

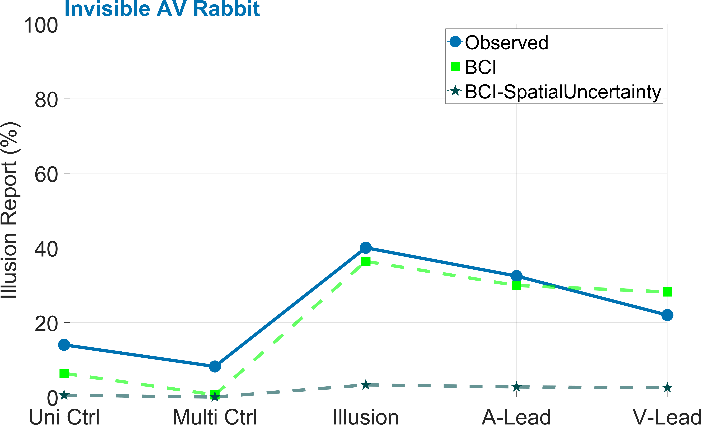
**

**Supplementary Figure 2. High auditory and visual spatial uncertainty in model simulations significantly diminish predicted illusion rates,** The group-averaged observed data (orange in the left and blue in the right panel) and the predicted illusion rates from the fitted BCI-Averaging (green) model and a model simulation with high spatial and auditory spatial uncertainty (i.e., BCI-SpatialUncertainty, teal).This BCI-SpatialUncertainty simulation was computed in the same way as the BCI model (See Methods), but with fixed spatial precision parameters σ_sA_ and σ_sV_ set to a high value of 15. In the regular BCI model, the σ_sA_ was fitted within the range of 0 to 5.84 and the σ_sV_ was fixed within the range of 0 to 0.84, as determined by the unisensory conditions' fit (See Supplementary Table 1). Uni Ctrl = unisensory control; Multi Ctrl = multisensory control; A-Lead = auditory lead asynchronous condition; V-Lead = visual lead asynchronous condition

**Supplementary Figure 3**


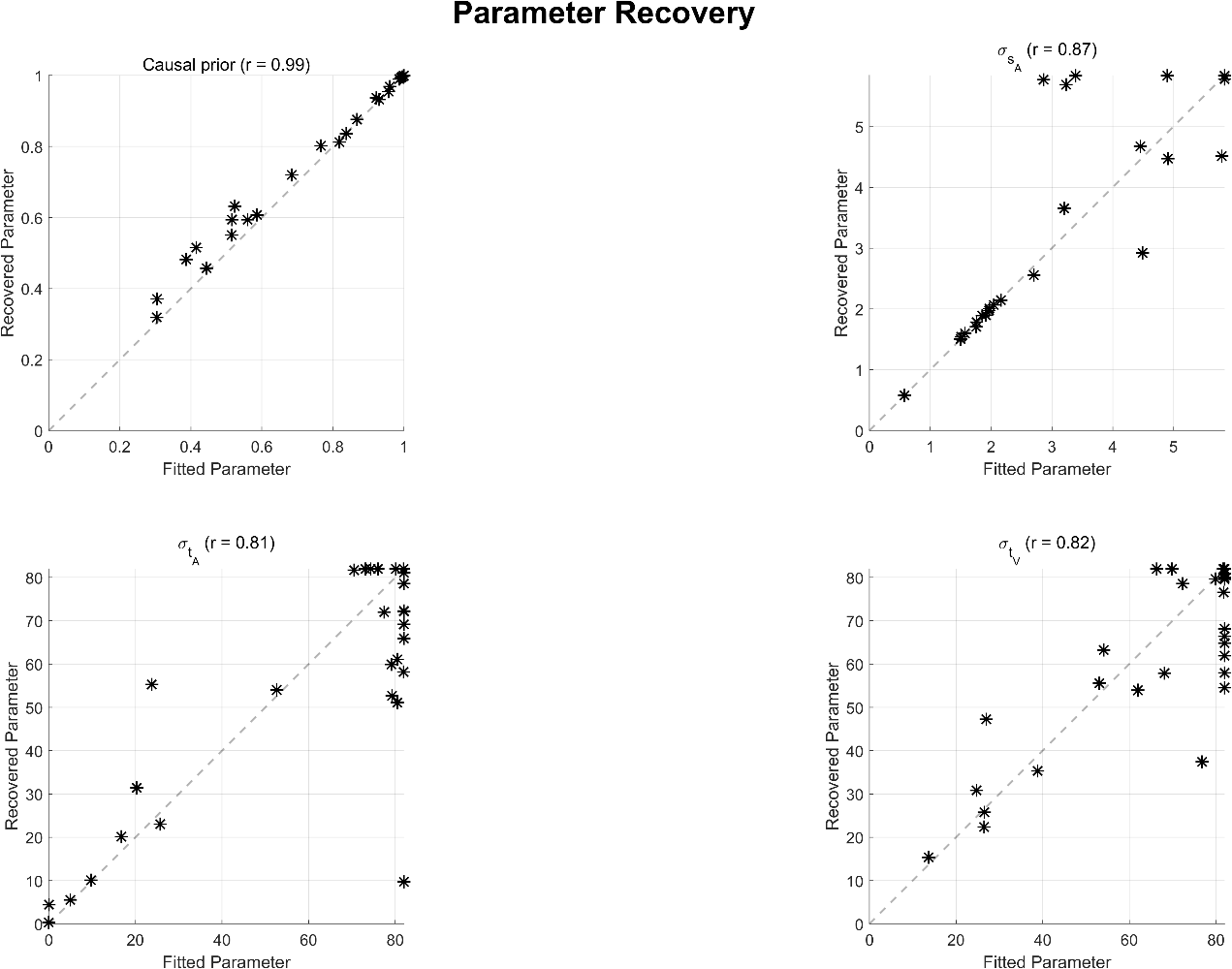


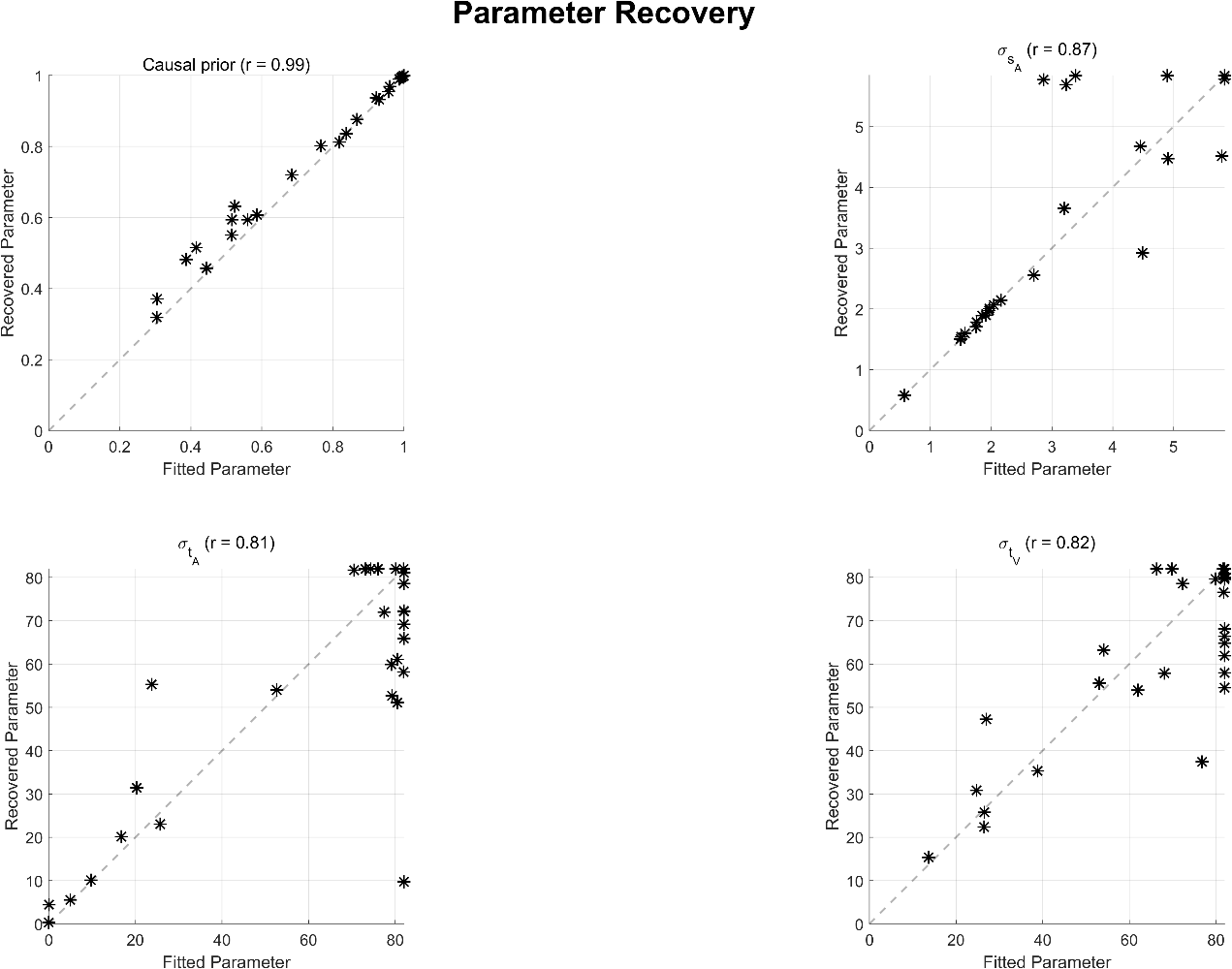

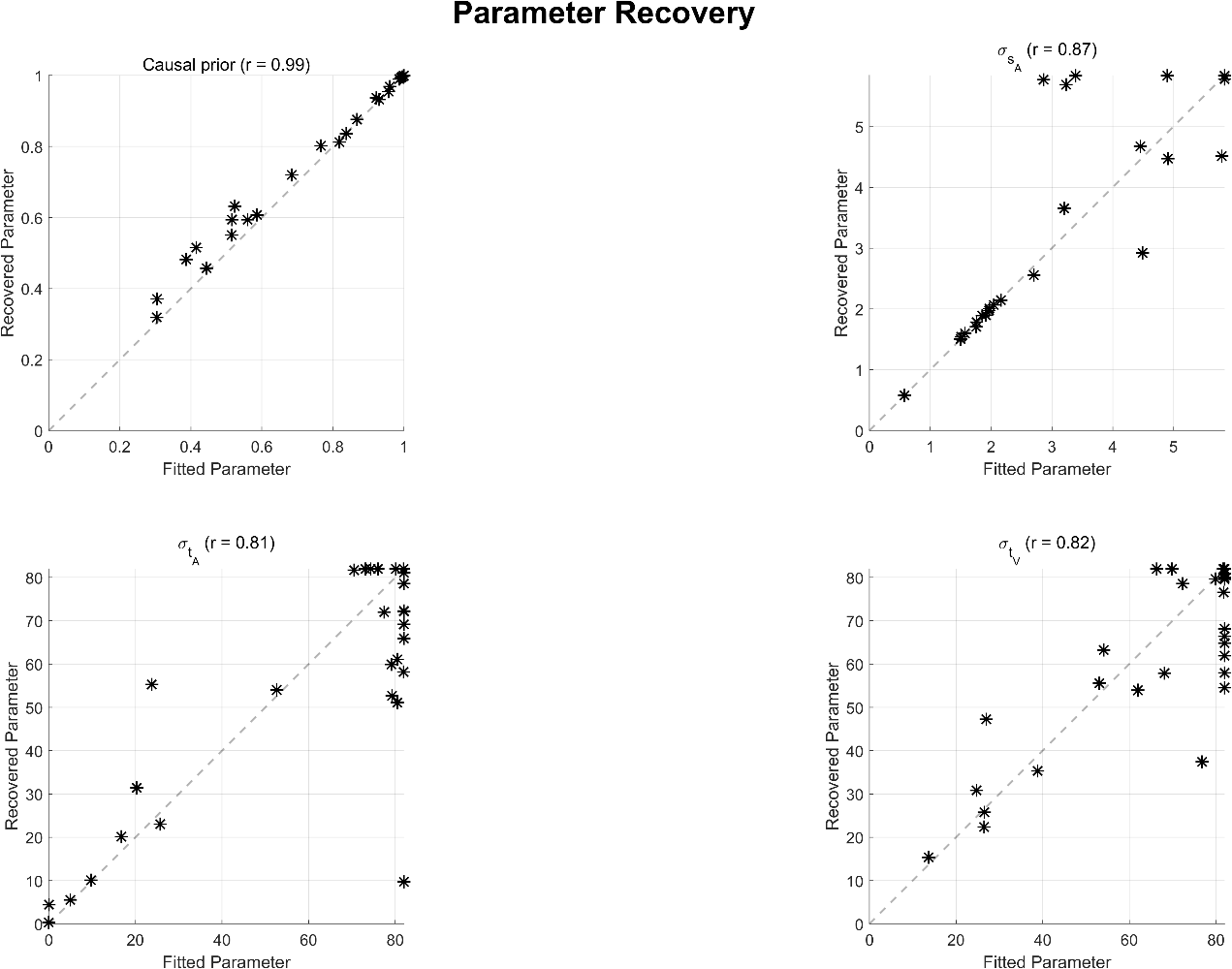


**Supplementary Figure 3.** **Parameter recovery for the fitted BCI model parameters (**p_common_, σ_sA_ , σ_tA_ and σ_tV_**)** Recovered parameter values vs. fitted parameter values for the parameters of the BCI model with the correlation coefficients. The black dashed line represents the unit line. Some parameters showed modest recovery, particularly (σ_tA_ and σ_tV_). This may reflect limitations in fitting a single global model across all 22 experimental conditions, which encompass two distinct illusions with different perceptual characteristics (See Methods).
